# Liver organoids: From fabrication to application in liver diseases

**DOI:** 10.3389/fphys.2022.956244

**Published:** 2022-07-18

**Authors:** Qianglin Liu, Anqi Zeng, Zibo Liu, Chunjie Wu, Linjiang Song

**Affiliations:** ^1^ School of Medical and Life Sciences, Chengdu University of Traditional Chinese Medicine, Chengdu, China; ^2^ Institute of Translational Pharmacology and Clinical Application, Sichuan Academy of Chinese Medical Science, Chengdu, China; ^3^ School of Pharmacy, Chengdu University of Traditional Chinese Medicine, Chengdu, China

**Keywords:** liver organoid, 3D culture, co-culture, disease modelling, drug screen

## Abstract

As the largest internal organ, the liver is the key hub for many physiological processes. Previous research on the liver has been mainly conducted on animal models and cell lines, in which not only there are deficiencies in species variability and retention of heritable material, but it is also difficult for primary hepatocytes to maintain their metabolic functions after *in vitro* expansion. Because of the increased burden of liver disease worldwide, there is a growing demand for 3D *in vitro* liver models—Liver Organoids. Based on the type of initiation cells, the liver organoid can be classified as PSC-derived or ASC-derived. Liver organoids originated from ASC or primary sclerosing cholangitis, which are co-cultured in matrix gel with components such as stromal cells or immune cells, and eventually form three-dimensional structures in the presence of cytokines. Liver organoids have already made progress in drug screening, individual medicine and disease modeling with hereditary liver diseases, alcoholic or non-alcoholic liver diseases and primary liver cancer. In this review, we summarize the generation process of liver organoids and the current clinical applications, including disease modeling, drug screening and individual medical treatment, which provide new perspectives for liver physiology and disease research.

## 1 Introduction

As the largest internal organ, the liver is the key hub for many physiological processes. It not only participates in the metabolism of major nutrients, but also has diverse roles in the regulation of the immune system and the decomposition of heterogeneous biological compounds (Trefts et al., 2017), including many drugs, and other functions. Therefore, liver disease has an enormous impact on human health. Liver disease causes approximately two million deaths worldwide each year (Asrani et al., 2019), and epidemiological studies have shown that the burden of liver disease varies among people of different ethnicities, genders, geographic regions, financial positions and social ladders. In addition, certain liver diseases also show genetic correlations. With the aging of the global population, the prevalence of obesity, diabetes and other diseases is gradually increasing, which also increases the incidence rates of non-alcoholic steatohepatitis and chronic liver disease (Estes et al., 2018).

**FIGURE 1:**
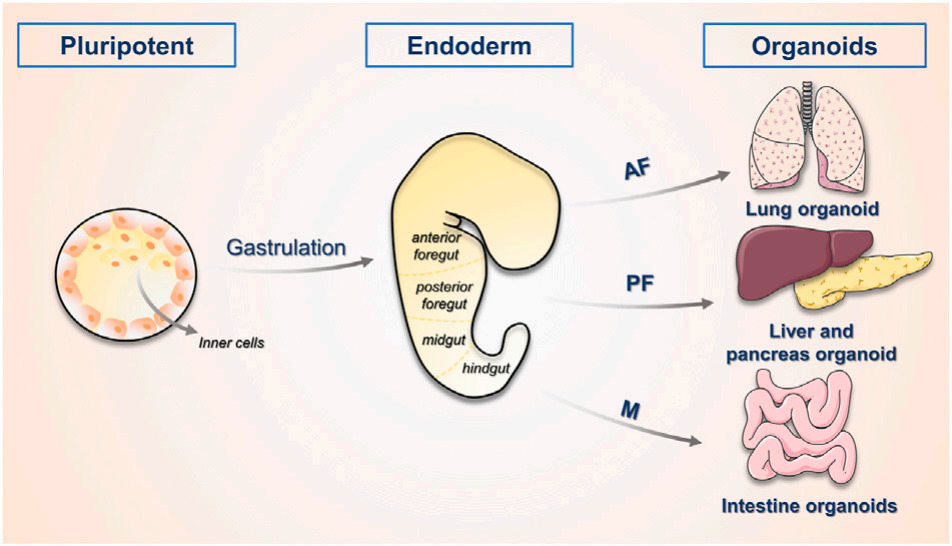
The process of organogenesis. The physiological process of human embryonic development begins with the oosperm, which divides several times to form the Morulaand then develops into the blastocyst. The inner cell mass within it serves as the early embryonic stem cells and has developmental omnipotence. The ectoderm, mesoderm and endoderm form various tissues and organs, this process is called organogenesis. The study of the process of organogenesis contributes to the directed construction of related organoids. AF, anterior foregut; PF, posterior foregut; M, midgut.

The development of the liver is first initiated by the inner cell mass, which progressively develops into an embryo. As illustrated in Figure 1, the posterior foregut of the endoderm develops into the liver as the triple germ layer forms. The liver is formed by the growth of the ventral foregut epithelium, which is first to develop into the hepatic bud structure. The hepatic buds generate hepatocytes and the cholestatic epithelium, while the adjacent mesenchymal stroma of mesodermal origin constitutes hepatic fibroblasts and stellate cells (Lancaster and Knoblich, 2014). The growth of the liver also involves the establishment of innervation, extensive vascularization and interactions between various types of cells, and it eventually develops into an organ with complex architecture and function. In an *in vitro* model, bile duct cells and hepatocytes complete cyclic renewal every 60 and 150 days, respectively (Magami et al., 2002). Although this is slower than the turnover rate of other organs of endothelial origin, the liver demonstrates a remarkable ability to regenerate after suffering injury.

To define the pathological changes of these diseases and discover the potential treatment approaches, researchers require an array of approaches to understand the development of liver structure and function, the pathogenesis of liver diseases, and the responses to related treatments. During the past few decades, most *in vitro* studies of chronic liver diseases have used two-dimensional (2D) cultured cell lines. Cell lines from benign or malignant tumours of the liver and primary human hepatocytes (PHHs) are the main sources for these *in vitro* studies because cancer-derived hepatocyte lines lack their normal liver cell counterparts, despite their ability to proliferate indefinitely. Most importantly, they lack most of the cell types that differentiate into primary tissues (Schutgens and Clevers, 2020). Although PHHs retain many of the characteristics of normal hepatocytes, their viability in culture is limited to a few days, so there is a constant need for fresh donor tissues, which are often difficult to obtain. In addition, their initial establishment involves not only extensive genetic but also phenotypic adaptation to culture conditions (Nuciforo and Heim, 2021). Two-dimensional cultured cell models, such as PHHs and induced pluripotent stem cells (IPSCs) also lose the polarity that hepatocytes exhibit *in vivo* because they are forced to adopt a flat morphology *in vitro* (Shulman and Nahmias, 2013).

By contrast, the *in vivo* models for studying the pathogenesis of liver-related diseases are mainly animal models (Aqeilan, 2020), which facilitates a greater and more sophisticated dimension of understanding. While animal models used to study liver disease mechanisms and targeted therapeutics share key biological and histological features with the liver in humans (Prior et al., 2019), the intrinsic differences between animal models and humans cannot be easily resolved, especially in replicating the key aspects of the complex structure and metabolic functions of the liver in humans, which has severely affected several potentially effective therapeutic agents in clinical trials. In addition, most of the animal models used in preclinical studies are based on inbred lines with genetic homogeneity that lack the heterogeneous genetic diversity of humans.

Organoids are established over long periods of time and maintain genetic sustainability, thereby achieving more complex structures similar to those of the mammalian body, and they have been applied to rodents (Kuijk et al., 2016), canines (Nantasanti et al., 2015; Gabriel et al., 2022), cats (Kruitwagen et al., 2017) and humans. In addition, while both *in vitro* models and organoids are capable of providing causal evidence of pharmacological and genetic targeting, the assay throughput of organoids is well beyond the maximum capacity of mouse models (Takebe et al., 2017). For the past few years, researchers have introduced transcriptomics, proteomics and metabolomics into studies of organoid cultures, promoting new discoveries and complementary investigations in 2D cultures and animal models. We summarize the advantages and disadvantages of different *in vitro* models in Table 1. In recent years, much progress has been made in establishing several different organoid culture protocols to simulate the human liver and model liver-related diseases. In addition, organoids have potential for drug testing and even organ replacement. Here, we describe the principal derivation procedures, as well as the classification of liver organoids, and discuss their present and emerging applications in disease modeling, drug screening, and regenerative medicine. Finally, we highlight some of the challenges that remain in the field.

**TABLE 1:** Comparison of different hepatic *in vitro* model systems.

Classifications	Sources	Advantages	Disadvantages	Reference
Animals	rodents, canines, cats, etc.	Experimental materials are relatively easy to obtain	Differences in structure and physiological state exist; lack the heterogeneous genetic diversity of humans	Broutier, et al. (2016)
				Kruitwagen, et al. (2017)
				Kuijk, et al. (2016)
				Magami, et al. (2002)
				Nantasanti, et al. (2015)
PHH	Liver tissue	Less experimental investment; Retaining genetic background; proliferate indefinitely	Lack of complexity of morphology; lose the polarity that hepatocytes exhibit *in vivo*	Huch, et al. (2013)
				Kang, et al. (2016)
				Nuciforo and Heim (2021)
iPSCs	Fibroblasts and others	Retaining genetic background	Lack of complexity of morphology	Asai, et al. (2017)
		High throughput screening	More experimental expenses	Coll, et al. (2018)
				Ohnuki and Takahashi (2015)
				Sampaziotis, et al. (2015)
				Schutgens and Clevers, (2020)
				Takebe, et al. (2013)
				Takebe, et al. (2017)
				Wang, et al. (2016)
Organoids	Adult liver, foetal liver, and pluripotent stem cells	Possesses a complex three-dimensional structure	Difficulty of the experiment process	Bao, et al. (2021)
		Preservation of gene stability and ability to perform genetic manipulation	More time and materials spent on the experiment	Broutier, et al. (2017)
				Clevers, (2016)
				Drost and Clevers (2018)
				Lancaster and Knoblich, (2014)
				Leite, et al. (2016)
				Mccauley and Wells, (2017)

PHH, Primary human hepatocytes; iPSCs, induced pluripotent stem cells.

## 2 Overview of the liver and organoids

### 2.1 Origin of liver organoids

In 1981, Evans and others established pluripotent stem cells from mouse embryos (Lancaster and Knoblich, 2014) and in 1988, the first human embryonic stem cell line was isolated and cultured from human blastocysts by Thomson et al. (1998) In 1987, Bissell and colleagues reported that extracellular matrix (ECM) extracts play an important role in epithelial organization into 3D ducts and conduits in a mammary model (Li et al., 1987). However, it was not until 2009 that Clevers and colleagues generated intestinal organoids from adult intestinal stem cells by 3D culture of stromal gels (Sato et al., 2009), which is believed to be the first organoid ever established *in vitro*.

The definition of an organoid is the collection of organ-specific cell types that develop from stem cells or organ progenitors and self-organize through cell sorting and spatially-restricted lineage commitment in a manner similar to that *in vivo* (Lancaster and Knoblich, 2014). Self-organisation, the basis for organoid establishment, is achieved through direct cell-cell interactions (Asai et al., 2017). The general ability of cells to separate and reorganise through the process known as “cell sorting” forms generations with histogenic characteristics that are identical to those *in vivo*. Self-organisation depends not only on the classification of cells, but also on the correct execution of genealogical decisions for progenitor cells, which includes the appropriate direction of stem cell division, the correlation of synchronous and non-synchronous divisions, and the migration of differentiated daughter cells to particular locations within a specific tissue (Lancaster and Knoblich, 2014).

### 2.2 Derivation methods for liver organoids

Organoids are based on the ability of cells to self-organise, which is influenced by the physical characteristics of the culture environment, the requirement for endogenous and/or exogenous signals and the starting cell type and system conditions (Rossi et al., 2018). Different parameter selections of these characteristics can ultimately affect the features and the extent of applicability of the organoid as a biological prototype system.

Until now, several approaches for the generation of liver organoids have been published. The current mainstream approaches for the generation of liver organoids involve a PSC-derived approach, including transdifferentiation (Sun et al., 2019), and an ASC-derived approach. The different approaches for the generation of liver organoids and their characteristics are described in further detail below.

## 3 Classification of liver organoids

**FIGURE 2:**
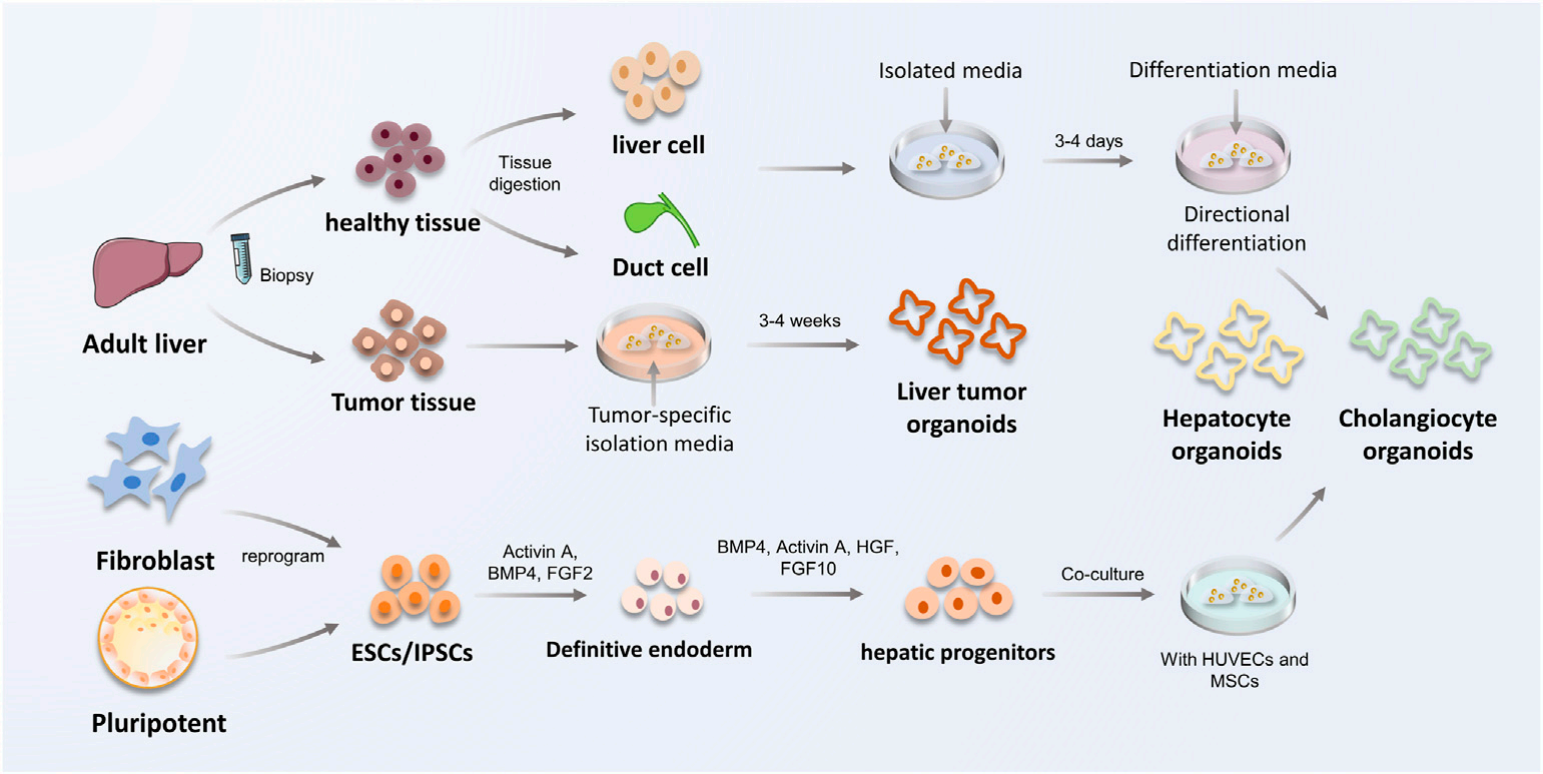
The construction procedure of ASC and PSC-derived liver organoids. The process of constructing liver organoids is divided into ASC and PSC origin. ASC are mainly derived from biopsy of adult tissues to obtain healthy or tumor tissues. Hepatocytes or bile duct tissues obtained from biopsies were inoculated in isolation medium, and subsequently hepatocyte organoids and cholangiocyte organoids were constructed using defined differentiation media. PSCs can be obtained by reprogramming fibroblasts *in vivo*, in addition to inner cell mass, which are capable to form hepatic progenitor cells by co-culture and special signal-mediated differentiation towards endoderm, and eventually obtain hepatocyte organoids and cholangiocyte organoids. HUVECs, vein endothelial cells; MSCs, mesenchymal stem cells.

Depending on the type of starting cell, organoids can be classified as PSC-derived or ASC-derived. Because each cell type originates at a different developmental stage, the maturity level of the organoid that can be derived from each type of starting cell is variable. The process of PSC- and ASC-derived organoid establishment is presented in Figure 2.

### 3.1 Pluripotent stem cells-derived organoids

Pluripotent stem cells (PSCs) are a type of cell that can self-renew and differentiate into specialized cell types that comprise one of the three germ layers *in vivo* (Liu et al., 2020). Thus, PSCs, which contain embryonic stem cells (ESCs) and induced pluripotent stem cells (IPSCs), are primarily used to study organogenesis and progression events that result in tissue generation (Schutgens and Clevers, 2020). PSCs are induced into hepatic endodermal cells by the action of activin A and then complete differentiation in response to developmental signaling pathways from FGF and BMP (Clevers, 2016).

IPSC generated by reprogramming of skin fibroblasts (Sun et al., 2019) can differentiate into all cell types in the body and precisely differentiate into different cell and tissue types under the control of induction signals (Asai et al., 2017; Mccauley and Wells, 2017). In addition, co-cultured with stromal cells, human umbilical vein endothelial cells (HUVECs) and human mesenchymal stem cells (MSCs), all of which have stem cell potential, can helped liver endodermal cells from human IPSCs (IPSC-HEs) recapitulate early organogenesis (Takebe et al., 2013; Rossi et al., 2018).

To address the lack of homologous substrate components, Ouchi et al. (2019) generated a foregut model by recapitulating early organogenic differentiation. The authors embedded foregut globules in a stromal gel, followed by co-culture with retinoic acid (RA) to directionally differentiate PSCs into foregut-derived organoids, thereby generating more complete organoids containing both epithelial components and mesenchymal cells which can differentiate into supporting lineages. Recently, in response to the disadvantage that PSC-derived organoids exhibit only immature fetal characteristics, Mun et al. (2021) used microbial short-chain fatty acids (SCFAs) to simulate changes in the microenvironment of the liver during postnatal development. This approach could increase albumin secretion and P450 activity, as well as the expression of hepatocyte genes (Aloia et al., 2019), which improved the metabolic ripening of IPSC-derived liver organoids.

### 3.2 Adult stem cells-derived organoids

Adult stem cells (ASCs) are undifferentiated lineage-committed cells. Terminally differentiated cholangiocytes or hepatocytes can also become activated by appropriate liver injury, which causes them to restart the cell cycle and induce liver regenerative repair by forming ASCs (Liu et al., 2020). However, other ASCs, such as hematopoietic stem cells, do not have this characteristic. Compared to PSC-derived organoids, ASCs can only differentiate into components associated with the organ or tissue from which they are derived. However, ASC-derived organoids are more stable at chromosomal and structural levels, while having a lower incidence of single base changes (Huch et al., 2015). This is due to the presence of inherited and epigenetic abnormalities in IPSC-derived organoids during IPSC reprogramming and organ differentiation, which occur infrequently in ASC-derived organoids (Ohnuki and Takahashi, 2015).

ASCs are located in a very specific microenvironment, namely, the stem cell niche, which ensures that the cells are able to renew themselves. Stem cell niches are functional domains within stem cell populations capable of controlling the dynamics of tissue homeostasis under different conditions (Vining and Mooney, 2017), and they communicate with stem cells through mechanical signals that regulate cell fate and steer their developmental processes with a high degree of plasticity (Voog and Jones, 2010). ASCs are usually obtained from isolated cells or dissected tissue fragments of normal or tumour tissues (Schutgens and Clevers, 2020). The cells of tissue origin used to establish liver organoids, including hepatocytes and duct cells, usually have a slow turnover rate but can exhibit a remarkable capacity for tissue self-renewal and damage restoration after liver injury (Aloia et al., 2019). ASC-derived liver organoids of hepatocyte origin are perhaps slightly superior to those of bile duct origin in terms of maturity and physiological relevance. However, there is still no alternative to cholangiocyte-based liver organoids, for example, to study the function and pathology of the bile duct. Meanwhile, bile duct-derived liver organoids are inoculated more efficiently than hepatocyte-derived liver organoids, with EpCAM + hepatic duct cell-derived organoids having a higher efficiency than hepatocyte-derived organoids, even in the presence of TNF-α (Peng et al., 2018). ASCs obtained from biopsy specimens, such as tumour tissues, allow the generation of patient-derived organoids (PDOs). Liver organoids derived from healthy tissues initially form a monolayer of epithelial structures, which transdifferentiate into pseudo-lamellar epithelial cells (Broutier et al., 2017), while tumouroids can directly recapitulate the features of the various tumour subtypes. Furthermore, they retain histologic, genetic and transcriptomic features to mirror the original tumour tissue (Li et al., 2019).

Following chronic liver injury, oval cells, a population of bipotent progenitor cells derived from biliary epithelial cells (BECs) in the Hering duct that emanates from the biliary tree, trigger regeneration by restoring both hepatocytes and cholangiocytes, suggesting that they are an alternative source for new cell therapies. Following acute liver injury, such as partial hepatectomy (PHx), total liver chemical-induced damage or infection (Broutier et al., 2016), mature hepatocytes respond by proliferating. Interestingly, no significant dedifferentiation to a progenitor state was observed during this process (Nuciforo and Heim, 2021) Based on the reparative mechanism of acute liver injury, mature hepatocyte-derived liver organoids can be generated directly, cultured and expanded *in vitro* for long periods of time (Hu et al., 2018).

The Wnt signaling pathway is comprised of extracellular development-related proteins, and this pathway is responsible for initiating sustained tissue renewal by promoting stem cell activity; otherwise, tissue renewal is impaired (Clevers et al., 2014). Lgr5, which is one of the target genes of Wnt, is a commonly used stem cell marker (Clevers, 2016). When CCl4 was used to induce liver injury, the damaged tissue showed increased expression of Axin2-LacZ16 compared to healthy tissue. Furthermore, Lgr5+ cells not only express multiple Wnt target genes but also have features of bi-potent liver progenitors, which allows them to achieve *in vitro* expansion (Huch et al., 2013).

## 4 Characteristics of the culture environment

Organoid differentiation of PSCs consists of several stages. First, the differentiation of IPSCs to the definitive endoderm is followed by the directed derivation to foregut progenitors, which is a crucial step, and finally in the presence of BMP and RPMI, among other factors, to hepatoblasts (Sampaziotis et al., 2015). Subsequently, hepatic progenitors with bidirectional differentiation potential can differentiate into hepatocytes or bile duct cells in different settings, a process that usually requires co-culture with mesoderm-derived stromal components embedded in ECM, which promotes three-dimensional (3D) growth and organoid formation.

### 4.1 Co-culture

**FIGURE 3:**
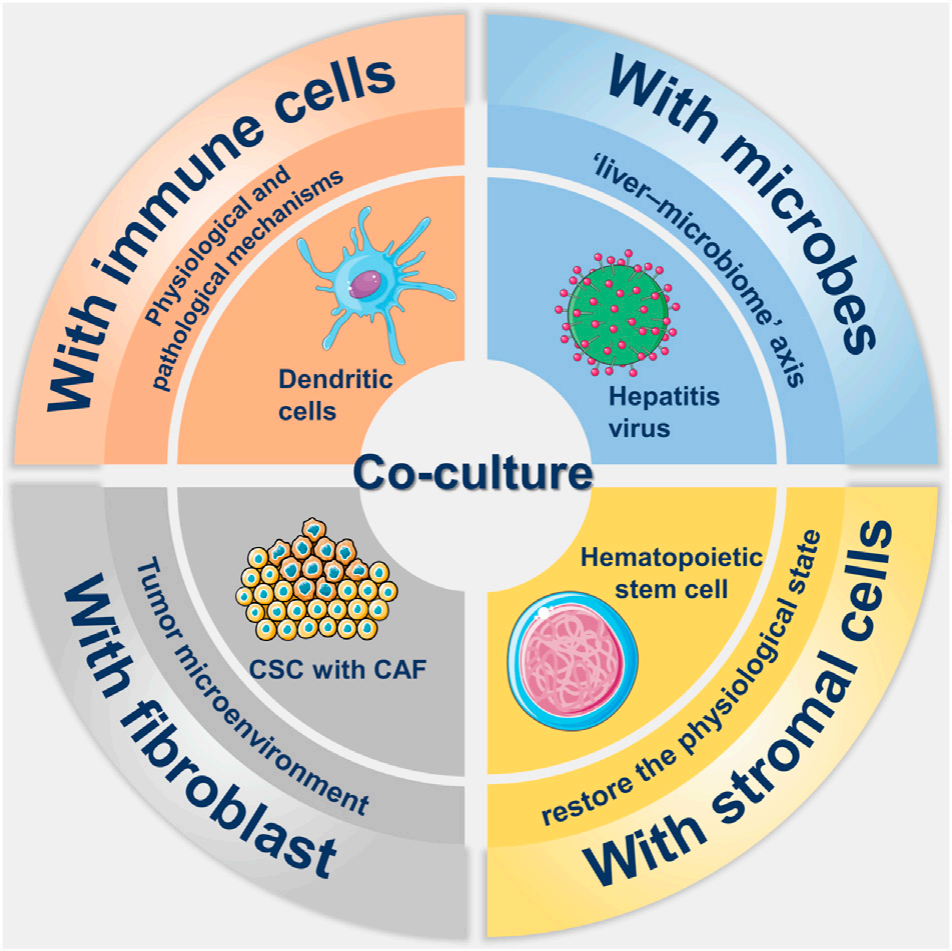
Overview about co-culture of liver organoids. There have been many researches in the field in co-culture of liver organoid, which mainly includes co-culture with immune cells, stromal cells, fibroblasts and microorganisms. The involvement of immune cells such as dendritic cells and stromal cells such as hematopoietic stem cells in co-culture helps to simulate the real environment *in vivo*. Co-culture with hepatitis virus not only reflects the role of the liver-microbe axis, but also allows for participation in disease modeling. Co-culture with cancer-associated fibroblast (CAF) in tumor modeling not only mimics the tumor microenvironment but also promotes the growth of cancer stem cells (CSCs).

As most liver organoids are typical of cystic organoids, containing only epithelial cell types, co-culture is needed to generate organoids. As displayed in Figure 3, co-culture methods are essential to study the interaction of organ tissues with immune cells, stromal cells, and fibroblasts, as well as to achieve the optimal reproduction of the actual environment *in vivo*.

#### 4.2.1 Organoids and immune cells

The co-culture of liver organoids with immune cells facilitates the exploration of the physiological state of the liver and the mechanisms of disease, in addition to promoting the investigation of the dynamic interactions between expanding tumours and immune systems (Bresnahan et al., 2020).

There are three types of systems in which organoids are co-cultured with immune cells (Bar-Ephraim et al., 2019). In the first system, immune-related cytokines are added to organoid media. In the second system, organoids are dissociated into single-cell suspensions and co-cultured with immune cells for a defined period of time before establishing organoids. In the third system, activated immune cells are co-cultured with organoids. However, of these three approaches, the first simply adds cytokines and lacks the relevant immune cell component, while the second is a co-culture of immune cells with organoids that have been digested into single cells, which does not prove that a co-culture system was established after digestion. By contrast, the third approach constitutes a putative co-culture system of immune cells. In addition, Yuki et al.(2020) described a method for establishing tumour-derived organoids, while preserving immune, stromal and mesenchymal cells from the same tumour specimen. The authors named this system “a native model,” which provides a holistic approach to the study of the tumour microenvironment and allows better simulation of tumour metastasis.

#### 4.2.2 Organoids and microbes

Many liver diseases, such as primary sclerosing cholangitis (PSC) and hepatic encephalopathy (HE) due to cirrhosis, have been reported to associate with microbial characteristics. For example, microbes play an essential role in liver inflammation, with the liver impacting and communicating with microbes through mediators such as bile acids or inflammatory signals. This connection is known as the liver-microbiome axis (Adolph et al., 2018). Therefore, the co-culture of organoids and microbial organisms can be used to study infectious or non-infectious liver inflammation.

The co-culture of organoids with viruses, which can mimic virus infection, has potential for infectious disease models. For example, Nie et al. (2018) generated an *in vitro* infection model with hepatitis B virus (HBV) after co-culture of PSCs with stromal cells on 3D microtiter plates, which developed into vascularised and functional tissue after transplantation. The significant release of viruses from infected IPSC-derived organoids in this assay and the high expression of the infection-promoting factors GPC5, PPARA and CEBPA in IPSC-derived liver organoids demonstrated the stability of this differentiated infection model.

During the COVID-19 epidemic, organoids were similarly applied to related research areas. Researchers reported that liver organoids were highly sensitive to SARS-CoV-2 infection and showed high expression of chemokines following infection, which is consistent with findings in lung specimens from human COVID-19 autopsies (Yang et al., 2020). In addition, co-culture of patient-derived organoids with microbes provides new insights on the roles played by microbes in the disease process (Mccarron et al., 2021). Liver organoids, as an emerging *in vitro* model, provide an effective way to investigate the human tissue response to SARS-CoV-2 infection.

#### 4.2.3 Organoids and fibroblasts

The co-culture of organoids and fibroblasts has been widely used in tumour patient-derived organoids. Cancer associated fibroblasts (CAF), which are derived from tissue-residual fibroblasts, endothelial cells and vascular smooth muscle cells, play an essential role in the establishment of the tumour microenvironment (Yamamura et al., 2015). CAFs can mold stem cell niches to maintain and facilitate the growth of cancer stem cells (CSCs) through direct contact with tumour cells or a paracrine pathway (Liu et al., 2021). The co-culture of CAFs with liver tumour-derived organoids promoted the growth of organoids, and the results showed that the effect of CAFs was achieved by expanding the volume of organoids instead of the number, although the effectiveness on organoid initiation was not confirmed.

In addition to the growth promoting effects on CSCs and liver tumour-derived organoids, CAFs can induce tumour drug resistance (Xiong et al., 2018). CAFs cause tumour drug resistance by affecting the tumour-specific microenvironment, including increasing tumour mesenchymal pressure and inducing vascular collapse. CAFs can also alter the immune response through ECM remodeling, thereby preventing the regulation of tumour tissue by immune cells (Liu et al., 2021). In addition to co-culture with organoids, co-transplantation of CAFs with organoids can result in more efficient tumour formation.

#### 4.2.4 Organoids and stromal cells

Hepatocytes and bile duct cells in the liver differentiate from the epithelium of the ventral foregut in the endoderm and the mesenchyme transforms from the mesoderm, so that hepatic progression represents a complex interplay between tissues of endodermal and mesodermal origins. To achieve this physiological state as much as possible, Takebe et al. (2013) generated functional vascularized liver buds by co-culture of IPSCs with stromal cell populations such as human umbilical vein endothelial cells (HUVECs) and human mesenchymal stem cells (MSCs). In addition to direct hepatocyte-stromal cell interactions, the stromal cell population also activates FGF and BMP through a paracrine pathway, which is necessary for the generation of 3D liver buds *in vitro*. However, due to the heterogeneity of IPSCs, common culture protocols do not guarantee that the cells used for organoid generation have the same characteristics, even if they come from the same cohort. To demonstrate the difference between the TGFβ1-induced artificial liver fibrosis model and the liver fibrosis caused by the natural development of NAFLD, Ali et al. established HepaRG-based bioengineered multicellular liver microtissues (BE-MLMs). HepaRG (cell line), HUVECs (human primary), KCs (human primary), and HSCs (human primary) were co-cultured in spheroid-laden hydrogels at different ratios while simulating liver tissue native configuration, such as nutrients/O_2_ gradients and direct cell-cell contacts between different cell types inside spheroids (Bao et al., 2021). Organoid of the HepaRG-based BE-MLMs is more complex and further describes the structural and metabolic environment of the liver. A new method for the co-culture of vascular endothelial cells and organoids was recently described by Pettinato and others. The authors reported that suppression of the Sonic hedgehog and Notch signalling pathways at an initial phase of differentiation significantly increased the expression of key proteins and the activity of enzymes (Pettinato et al., 2021).

Overall, co-cultures play an irreplaceable role in the construction of organoids, bridging the cellular and structural deficiencies of single germ layer-derived organoids in immune, neural and vascular aspects. In terms of disease modeling, co-cultured organoids can personalize the simulation of the patient’s *in vivo* environment and construct more effective disease models. However, there are differences between HUVEC and mature hepatic sinusoidal endothelial cells, which may lead to differences in subsequent applications for disease modeling. In addition, the current co-culture of TME components other than immune cells and fibroblasts with organoids still needs to be improved to further enhance the effectiveness of organoids.

### 4.2 Culture environment of liver organoids

#### 4.2.1 Matrigel method

The two-dimensional (2D) culture of hepatocytes primarily consists of monolayers on collagen gels and cultures covered with matrix or collagen gels, which are defined as “sandwich cultures” (Peng et al., 2021), but hepatocytes cultured in this way rapidly exhibit defects in their morphology. In organoid cultures, hepatocytes are mainly co-cultured with mesenchymal cells and supported by the matrix gel, which induces the aggregation into 3D spheroids. Table 2 compares and summarizes the characteristics of biomaterials from matrigel sources and non-matrigel sources.

**TABLE 2:** Comparing the differences between different organoid construction biomaterials.

	Sources	Biomaterial	Existing research
MATRIGEL	Natural	EHS tumor tissue	widely used for studies on cell differentiation, angiogenesis, and tumor growth
MATRIGEL-FREE	Natural	Alginate	supported differentiation and maturation of the organoids
		Collagen gels	inducing fibroblast differentiation during matrix remodeling
		Hyaluronic Acid (HA)	Improved cell-cell and cell-ECM interactions
		Silk	High stromal cell infiltration in the silk scaffolds
	Synthetic	PEG (Poly ethylene Glycol)	Improved growth and expansion of the organoids
		PLGA (Poly Lactic Glycolic Acid)	Improved wound healing
		PCL (Poly Caprolactone)	Improved tumoroid formation with porous PCL substrate
		Hybrid hydrogels	Low immunogenicity

EHS, Engelbreth-Holm-Swarm; ECM, extracellular matrix.

Matrigel is an extract from Engelbreth-Holm Swarm mouse sarcoma with components similar to those of a true basement membrane that can form a hydrogel at temperatures of 30°C and above (Benton et al., 2014; Aisenbrey and Murphy, 2020), and the effectiveness of matrix gel-based cultures for organoid generation has been widely demonstrated (Guan et al., 2017; Giobbe et al., 2019). However, the generation of Matrigel-based liver organoids suffers from a lack of cell function and maturation. Furthermore, the complex and uncertain composition of Matrigel makes it difficult to precisely demonstrate its role in organoid generation.

#### 4.2.2 Matrigel-free methods

The matrix gels prepared by decellularization, as described above, are of animal origin, and their residual animal protein content may induce an immune response in the host (Giobbe et al., 2019; Kozlowski et al., 2021). Therefore, the advent of chemically-synthesized matrices has remedied this shortcoming. A purely chemically-synthesized matrix that integrates key ECM proteins found in the liver, such as type IV collagen and fibronectin, was prepared using poly ethylene glycol (PEG) hydrogels as the skeleton and successfully used to culture liver organoids. In addition to the benefits of complete chemical synthesis and avoidance of immunological factors, the stable cross-linking of the PEG gel matrix allowed for the stable culture of liver organoids for more than 14 days (Sorrentino et al., 2020). In addition to purely chemically-synthesized substrates, the bioplotted poly-L-lactic acid scaffold (Wang et al., 2016), which permitted cells to develop in three dimensions and form cell-cell connections, was applied by Wang and colleagues. The cells grown within the ECM scaffold had markedly higher P450 activity and metabolizing enzymatic activity compared to IPSC-derived hepatocytes grown in a 2D matrix gel.

#### 4.2.3 Liver on-chip

With the development of organoid chips in recent years, liver chips have received much attention and investigation. The types of cells and culture approaches used for liver microarrays are constantly improving, and the overall model can be classified as simple microwell microarrays (Wiedenmann et al., 2021), complex microfluidic microarrays (Ya et al., 2021) and body on chip (Tao et al., 2021), a collection of multiple organoids. Microfluidic organoids not only enable more precise control of culture conditions, but also reduce the influence of human factors by imposing physical and mechanical controls (Telles-Silva et al., 2022). Based on the standardized nature of organoids, this model implies more consistent drug responses and more reliable results.

The liver organoids generated of the rat, dog and human involved using micro-engineered organoids containing primary hepatocytes and stromal cells. Primary hepatocytes of the three sources were located in the porous membrane with two parallel microchannels within the ECM sandwich, while stromal cells were located on the other side of the sandwich. Ya et al. (2021) used engineered liver lobule canines (LLC) to generate liver organoids. The addition of an oxygen concentration regulation chip (ORC) on top of this could more accurately reflect the blood supply and blood oxygen content of the hepatic arterial and venous systems in the actual liver. The advantages of this organoid over those cultured in a matrix gel are that it solves the issue of vascularization and the establishment of a naturally perusable hepatic sinusoidal system, which prolongs the application of the liver organoid.

#### 4.2.4 Other biomaterials methods

*In vitro* cells self-organizing into organoids are limited by the lack of circulatory system as well as oxygen and nutrients, which leads to the appearance of central organoid necrosis. To address this problem, vascularization methods have been applied to the construction of organoids, and interspersing fibers of endothelial cells between fibers containing parenchymal cells can facilitate vascularization and anastomosis with the host tissue *in vivo*. In addition to this, 3D printing techniques have been applied to construct vascularized frameworks for tissue development and differentiation of organoids in response to endothelial-parenchymal interactions (Leong et al., 2013).

Biomaterial scaffolds are mainly used for compartmentalization or restriction during organoid construction and are important for determining the final organoid structure and function. For example, the initial arrangement of epithelial and mesenchymal cells that initiate the development of ectodermal appendages in co-culture can be constructed by 3D modeling, which later consists of multiple parallel adjacent fibrous compartments filled with epithelial and mesenchymal cells as a whole (Pan et al., 2013).

## 5 Validation of liver organoids

After the liver organoid is generated, its performance can usually be assessed *in vitro* by morphological tests (Broutier et al., 2016; Aloia et al., 2019; Ogoke et al., 2022), functional tests (Akbari et al., 2019), such as P450 activity assays, and single-cell sequencing (Elbadawy et al., 2020). Compared to morphological and functional tests, single-cell sequencing is more reflective of the similarity between the organoid and the primary liver. The Human Cell Atlas Project is a worldwide study that characterizes cellular and tissue components through a single cell genomics approach, and it meticulously identifies different cells and tissues and defines all cell types in the human body based on their unique molecular profiles (Lindeboom et al., 2021). Single-cell transcriptomics also plays an important role in the validation of organoids. A single-cell genomic approach can quantify the similarity of cells within liver organoids to the primary liver tissue, and this approach offers superior resolution and accuracy compared to bulk RNA-sequencing (Brazovskaja et al., 2019; Shinozawa et al., 2021). Camp et al. (2017) reported the regeneration of PSCs to a hepatocyte lineage by applying single-cell RNA sequencing at a 2D level, and a 3D liver bud was generated by co-culture of hepatocytes and stromal cells. Furthermore, the similarity to fetal hepatocytes was demonstrated based on the results of single-cell RNA sequencing, thereby proving the validity of the liver organoid.

## 6 Application of liver organoids

**FIGURE 4:**
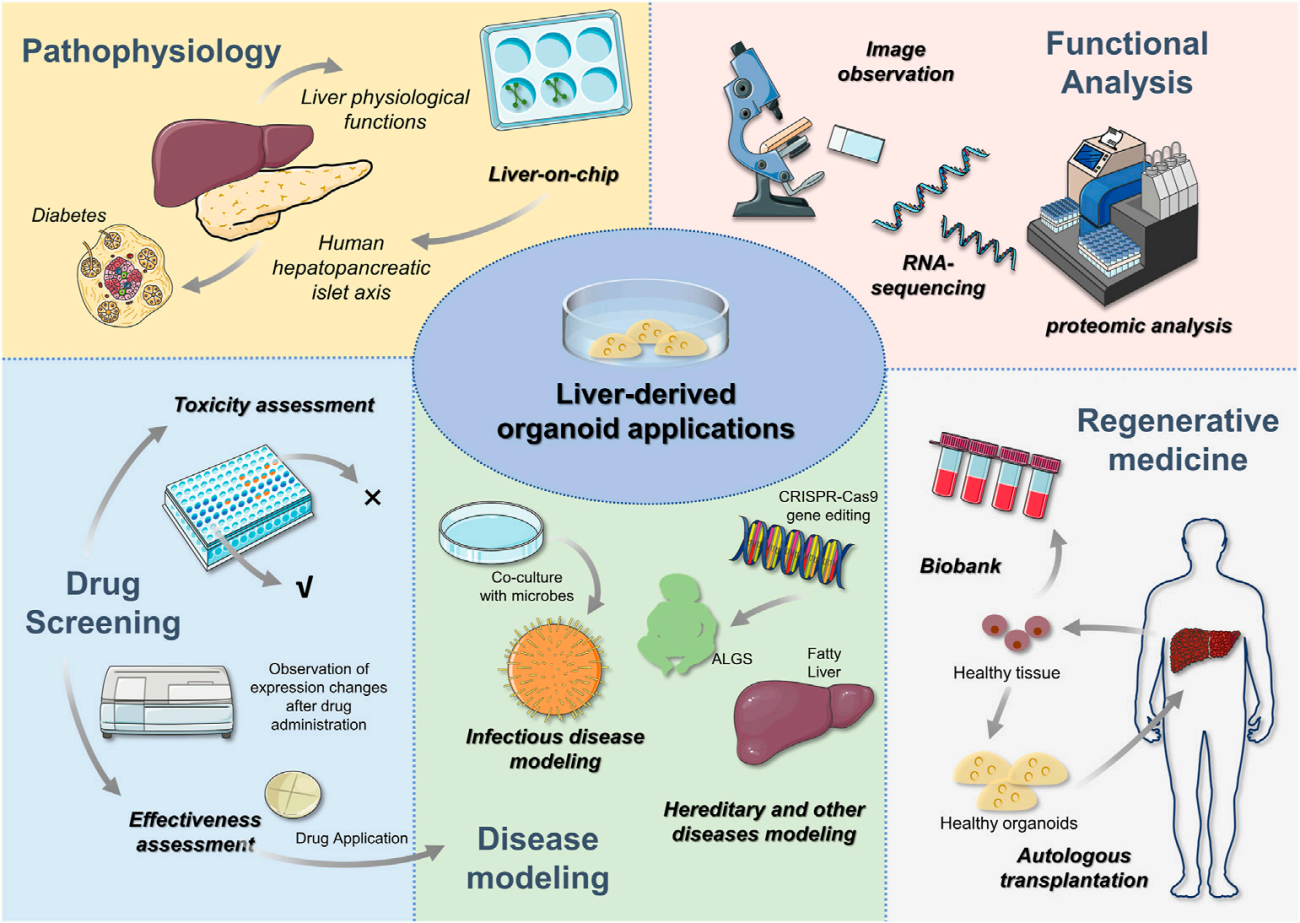
Liver-derived organoid applications After the liver organoid has been constructed, relevant expressions can be examined by morphology, RNA sequencing and proteomic analysis to determine the validity of the organoid. Subsequently, liver organoids can be used for liver pathophysiology research, disease modeling, drug screening and regenerative medicine. In addition to further understanding of liver physiology, *in vitro* 3D organoids can be used to construct multi-organ systems to study the role of different organs through liver-on-chip. For example, the role of hepatopancreatic islet axis in diabetes. Drug analysis includes efficacy and toxicity analysis, which contributes to the clinical application of drugs. Disease modeling is a popular area for liver orgaoids applications. Congenital and genetic diseases have been constructed through gene editing techniques. The availability of liver organoids also offers the hope to autologous organ transplantation. Healthy liver tissues in patients are able to be expanded and then transplanted, which can avoid the emergence of anti-host reactions. In addition to this, biopsied liver tissue is able to expand the biobank.

Existing disease models, such as cancer models, only reproduce a patient’s tumour, and the lack of a functional holistic component in these models leads to limitations in their application. Commonly used animal models, while contributing to the fundamental field of disease research, can require long culture and experimental periods and often do not adequately reflect the histological complexity and genetic specificity of the human body (Drost and Clevers, 2018; Nuciforo and Heim, 2021). By contrast, organoids can serve as preclinical models because they are physiologically and functionally similar to their primary organs. As such, the study of disease is not only limited to basic theory; instead, it also contributes to the development of clinical therapeutics, which is superior to other established 2D models. The current use of liver organoids is summarized in Figure 4.

### 6.1 Disease modelling

The sources of the disease models used to generate organoids can be classified as patient-derived organoids and CRISPR + organoids. Patient-derived normal or abnormal specimens preserve the genetic background of the respective individual, and thus, are important in disease modelling of monogenic diseases and cancers, as patient-derived tissues include specific genetic mutations. In addition, they can be used to develop individualized drug tests or treatment plans for patients (Nuciforo and Heim, 2021).

CRISPR-Cas9-mediated genome engineering is one of the most popular gene editing technologies, and it mainly includes gene knock-in and knock-out techniques. After CRISPR-Cas9-induced double-strand breaks occur, the induced breaks can be repaired by non-homologous end joining or homology-directed repair (Zhan et al., 2019; Hendriks et al., 2020). Compared to patient-derived organoids, CRISPR + organoids can overcome the limitation of the sample source. In addition, after oncogene knock-out (Artegiani et al., 2019), it is possible to continuously observe the changes in the organoid and to further intervene in various stages of the disease. In addition to disease modelling, genome-scale CRISPR screening can identify new genes for cancer research (Zhan et al., 2019; Ringel et al., 2020).

#### 6.1.1 Genetic diseases

Alagille syndrome (ALGS) is an autosomal dominant disorder that results in multiple organ abnormalities due to mutations in *JAG1*, which blocks the Notch signalling pathway. The main hepatic lesion is chronic cholestasis due to the lack of intrahepatic bile ducts (Turnpenny and Ellard, 2012). In a study by Huch et al. (2015), structural defects in the biliary tract were generated using biopsies derived from patients to model ALGS disease. The inhibition of nicotinamide, R-spondin, etc., was found to lead to a reduction in biliary marker expression and biliary cell apoptosis in ALGS patient-derived organoids. Guan et al. (2017) prepared iPSCs using fibroblasts from patients and generated IPSC-derived liver organoids expressing *ALGS1*. The pathogenic *ALGS1* mutation C829X was introduced and restored in IPSC-derived liver organoids and controls by CRISPR-Cas9 genome editing, and it was found that bile duct formation in liver organoids was increased following reversal of the *ALGS1* mutation. The efficiency of organoid formation and biliary transit function were also closely associated with the *JAG1* mutation.

Citrullinemia type 1 (CTLN1) is a relatively rare inherited metabolic disorder that causes severe and fatal neonatal hyperammonemia. It is mainly due to a mutation in arginosuccinate synthetase (ASS1) that affects the detoxification function of the liver, thereby blocking the urea cycle and causing a significant accumulation of ammonia in the body that cannot be converted to urea, ultimately leading to hyperammonemia (Nuciforo and Heim, 2021). Research into this disease is hampered by the high clinical variability of the disease and the lack of models that can predict the severity of the phenotype early in the course of the disease. Akbari et al. (2019) performed disease modelling of CTLN1 by generating liver organoids using biopsies from patients. The expression of *ASS1* was not detected in patient-derived organoids, but it could be detected in healthy donors, indicating the importance of *ASS1* defects in CTLN1. The ability to reverse disease-associated ammonia accumulation by genetic manipulation techniques to achieve the expression of *ASS1* suggests that the model is suitable for genetic manipulation. This study also provides early evidence for the role of organoids in genetically-corrected treatments for genetic disorders.

Mitochondrial DNA deficiency syndrome (MDS) is a serious genetic disorder caused by iron overload in the body, which is mainly due to mutations in *DGUOK*. The liver, as the main storage site for iron in the body, is more sensitive to the oxidative stress caused by iron overload, which ultimately leads to severe liver damage. However, the process from iron overload to liver failure is not known. Recently, Guo et al. (2021) used IPSCs from patients carrying a *DGUOK* mutation to generate liver organoids *in vitro*, a model that not only has the characteristics of hepatocytes, but also includes the patient’s genetic context. In addition, corrections by CRISPR/Cas9 gene editing enable more accurate controls. The ability of n-acetylcysteine (NAC) to reduce the sensitivity of NCOA4-dependent ferritin degradation-mediated iron pendant disease in lysosomes to iron overload has led to the further study of MDS.

#### 6.1.2 Alcohol-related and non-alcoholic fatty liver diseases

Alcoholic liver disease (ALD) is a liver disease that is closely related to alcohol use. Chronic alcohol abuse (>60 g/day) plays an important role in the development of the disease, and genetic factors, such as patatin-like phospholipase domain containing 3 (PNPLA3) and transmembrane six superfamily member 2 (TM6SF2) expression, viral infections, obesity or malnutrition, are also associated with ALD (Lackner and Tiniakos, 2019). ESC-derived liver organoids co-cultured with human fetal liver mesenchymal cells can mimic the pathophysiological and developmental stages of the disease, including oxidative stress and inflammatory-mediated fibrosis. This disease model can also be used for the study of steatohepatitis that follows ALD. As this model shows significant long-term expansion potential over 20 generations, it can be applied as an industrial-scale model for studying the potential mechanisms of the disease, screening of drugs and conducting research related to ALD prevention and treatment (Wang et al., 2019).

Unlike ALD, the causative factors for non-alcoholic fatty liver diseases (NAFLD) may include high-energy diet, sedentary lifestyle, obesity, diabetes and hyperlipidemia (Younossi et al., 2019). When liver organoids were exposed to free fatty acid, hepatocytes showed steatosis and ballooning, with increased levels of pro-inflammatory cytokines and collagen, demonstrating the role of inflammation and fibrosis in the disease, and thus, recapitulating key features of non-alcoholic steatohepatitis (NASH) (Soret et al., 2020). Patient-derived organoids are capable of modeling specific diseases, while patient-derived NASH organoids show strikingly diverse transcriptomes and functions, including increased growth kinetics, lipid accumulation and sensitivity to apoptotic stimuli. The co-culture of NASH organoids with hepatic stellate cells, T cells and Kupffer cells can effectively validate NASH-related inflammation, fibrosis and tumour development. As such, this approach more accurately reflects the hepatic microenvironment of NASH patients than the NASH model constructed from healthy liver organoids (Mccarron et al., 2021). The upregulation of some novel genes in NASH organoids (Elbadawy et al., 2020) may provide a possible pathway for the use of liver organoids in the diagnosis and treatment of NASH in the future.

#### 6.1.3 Infectious diseases

Viral hepatitis, mainly caused by hepatophilic viruses, causes acute hepatocyte necrosis, degeneration and inflammation. Hepatitis B virus (HBV), which causes chronic infection in more than 200 million individuals worldwide, is the predominant infectious agent of the liver (Wose Kinge et al., 2020). HBV exhibits significant heterogeneity, including clinical manifestations ranging from self-limiting infection to cirrhosis or hepatocellular carcinoma development, and different outcomes in response to the same drug therapy. This has led to the difficulty in providing a complete overview of the HBV replication cycle and the deficiency in representing individualized genetic backgrounds in the study of related diseases. By co-culturing IPSCs with mesenchymal and endothelial cells, IPSC-derived organoids are a good model for HBV research, which encompasses patient-specific genetic backgrounds and susceptibility to HBV infection compared to IPSC-derived hepatic-like cells (Nie et al., 2018). In addition to healthy liver organoids, the use of HBV-infected chronic cirrhotic liver tissues to generate HBV-infected patient-derived liver organoids enables the study of HBV development. Despite the lack of phenotypic evidence for abnormal growth, HBV-infected non-tumourigenic patient-derived liver organoids exhibit early cancer gene profiles (De Crignis et al., 2021), which may provide an approach for the early personalized treatment of HBV-associated hepatocellular carcinoma (HCC).

Due to the ACE2+/TMPRSS2+ expressing cell population in human bile ducts, human liver ductal organoids can be used for SARS-CoV-2 disease modelling. By co-culturing with SARS-CoV-2, human liver ductal organoids exhibited susceptibility and supported robust viral replication (Zhao et al., 2020; Lui et al., 2022). In addition to healthy human liver ductal organoids, patient-derived organoids can respond to the relationship between different diseases. For example, NASH-derived organoids demonstrate the permissible to SARS-CoV-2 pseudovirus, providing a possible explanation for the severe outcome of COVID-19 in NASH patients (Mccarron et al., 2021).

#### 6.1.4 Liver fibrosis

Hepatic fibrosis is a dynamic pathological process that essentially results from the excessive accumulation of heterologous hepatic myofibroblastic components, leading to abnormal production of connective tissue in the liver (Parola and Pinzani, 2019). Initially, chronic inflammation in the liver occurs in response to various pathogenic factors such as hepatitis virus infection, fatty liver disease, immune disorders and drug or chemical exposure. Inflammation primarily activates hepatic stellate cells within the liver interstitium, which is the central event in liver fibrosis. Furthermore, hematopoietic stem cells are activated and differentiate into myofibroblasts, whose increase leads to the massive accumulation of ECM (Bao et al., 2021). The chronic inflammatory process also produces several immune cells that are driven by inflammatory factors, which damages hepatocytes and causes them to lose their robust regenerative capacity, ultimately leading to the development of irreversible cirrhosis.

Three-dimensional liver organoids were constructed by co-culture with HepaRG and hepatic stellate cells. After exposure to allyl alcohol and methotrexate, the organoids showed enhanced activity of hepatic stellate cells and secretion and deposition of collagen, providing evidence for the establishment of a fibrosis model based on HepaRG-derived liver organoids. In addition, acetaminophen use results in hepatocyte injury-dependent activation and collagen production in hepatic stellate cells, while histone deacetylase suppression prevents activation in these cells (Leite et al., 2016). One of the clinical manifestations of autosomal recessive polycystic kidney disease (ARPKD) is congenital hepatic fibrosis. The generation of liver organoids with a common pathogenic mutation in *ARPKD* demonstrated that the increased TGFβ expression generated by this mutation in bile duct cells could stimulate myofibroblasts to form collagen fibers (Guan et al., 2021), which ultimately resulted in the development of liver fibrosis.

#### 6.1.5 Cancer

ACS-derived liver organoids and patient-derived xenografts (PDXs) are common sources of 3D *in vitro* liver tumour models. However, PDXs associate with a low implantation rate, and thus, they have limited application in disease modelling (Cavalloni et al., 2016). Patient-derived tumour organoids have been shown to retain the histology, gene expression and oncogenic potential of the original tumour based on long-term *in vitro* expansion, and these features can also be seen in patients after transplantation (Broutier et al., 2017).

Li et al. (2019) generated PDOs from tissues of different regional biopsy sources and investigated the effects of 129 FDA-approved drugs, demonstrating the roles of intra- and inter-patient factors in drug response heterogeneity, which may explain why promising drugs succeed in preclinical models but fail in clinical settings. Liver tissues from tumour patients obtained by needle biopsy, including tissues from various clinical stages of HCC of different etiologies (Nuciforo et al., 2018), have been used to construct HCC organoids. While retaining the biomarkers of the original tumour, HCC organoids are significantly different morphologically from those of non-tumour derived organoids. HCC organoids, which more closely resemble the tumour-specific microenvironment of the original tumour, may be used to further investigate individualized therapies. However, tumour-derived organoids, such as HCC organoids, are affected by the overgrowth of healthy contaminant-derived organoids, which may be due to differences in genetic stability. To avoid the growth of healthy contaminant-derived organoids, the time of tissue digestion should be increased (Broutier et al., 2017). Furthermore, the outcome of organoid generation correlates with the degree of differentiation of the original tumour, with only one quarter of HCC biopsies progressing to organoids and those are usually derived from undifferentiated specimens (Nuciforo et al., 2018).

Current sources of organoid models for Cholangiocarcinoma (CCA) include biopsy tissue from CCA patients and genetically engineered human cholangiocyte, and have been useful in disease metabolism studies, drug screening and individualized medicine (Massa et al., 2020). BRCA1-associated protein 1(BAP1), a tumour suppressor, was introduced into human cholangiocyte organoids by CRISPR-Cas9 using a loss-of-function technique, and the organoids were injected into mice subcutaneously, as well as into the liver, after editing to demonstrate tumourigenic ability. After adding doxorubicin to induce BAP1 expression, the morphology of the organoid gradually reverted to a monolayer (Artegiani et al., 2019), demonstrating that BAP1 plays an important role in the development of cholangiocarcinoma. By manipulating classical genes, such as *P53*, *RB*, *MYC2* and other oncogenes, Sun et al. (2019) demonstrated that oncogenesis in hepatocytes could be prevented by inhibiting both Notch and JAK-STAT signalling pathways. By RNA sequencing of co-cultured and monocultured cells, the results showed upregulation of gene expression associated with TNF signaling. Hepatocellular carcinoma cell-endothelial vascular secretory signaling in the co-culture model differentiated macrophages toward a pro-inflammatory and pro-angiogenic phenotype, suggesting that endothelial cells induce an inflammatory microenvironment in hepatocellular carcinoma cells by recruiting immune cells (Lim et al., 2022).

### 6.2 Drug screening

#### 6.2.1 Effectiveness assessment

Polycystic liver disease is a type of cystic lesion caused by the presence of fetal cholestatic cells in the liver (Raynaud et al., 2011), which leads to a dominant effect in the bile ducts, thereby increasing fluid secretion into the lumen, enhancing bile duct cell proliferation and leading to impaired liver function. Bile duct organoids were used to examine the effects of drugs on shrinking cysts in the treatment of polycystic liver disease. Octreotide, a synthetic analogue of a growth inhibitor, has been used in clinical settings to reduce the size of cysts in polycystic liver disease by eliminating the effects of secretin on cystic lesions (Sampaziotis et al., 2015).

Hepatic lipid deposition is a unique and common condition in cats in which fats are broken down into free fatty acids and then transported through the bloodstream to the liver, where they accumulate as triacylglycerols in hepatocytes. Using a feline liver organoid system to examine the effects of drugs on treating hepatic lipid deposition in cats, Haaker et al. (2020) validated the effects of AICAR, T863 and PF 06424439 on hepatic lipid deposition by quantitative TAG assays, lipid droplet staining analyses and quantitative polymerase chain reactions to further provide evidence for *in vitro* studies of hepatic lipid deposition.

In addition, traditional Chinese medicines play important roles in the treatment of liver diseases. After applying cholesterol + MIX (mainly cholesterol and other small molecules), Wu et al. (2019) showed increased expression of mature hepatocyte and bile duct cell markers in hepatobiliary analogs, which also showed improved drug metabolism, glycogen storage capacity and Alb secretion capacity compared to analogs in the absence of the medicine, demonstrating the positive benefits of herbal medicines in liver health.

#### 6.2.2 Toxicity assessment

As the toxicity of certain drugs, such as the proprietary compound JNJ-2, can associate with significant species variability, liver organoids provide a considerably more accurate tool for drug toxicity testing than animal models (Jang et al., 2019). For drug-induced liver injury, the applicability of more than 200 marketed drugs and their resulting cholestatic effect and/or mitochondrial toxicity has been assessed by high-throughput liver organoid models (Shinozawa et al., 2021).

Liver organoids are also used to assess drug toxicity in cells of different origins. For example, Kang et al. (2016) established ESC-, PSC- and primary hepatocyte-derived organoids. The hepatotoxicity of acetaminophen (AAP) and aflatoxin B1 (AFB1) was evaluated, and it was found that AAP decreased cell viability and increased lactate dehydrogenase activity in a dose-dependent manner in all cell types. Furthermore, AFB1 showed different dose-dependent responses depending on the cell type and rapidly decreased cell survival in primary hepatocyte-derived organoids.

#### 6.2.3 *In vitro* studies of liver physiology and regenerative medicine

The properties of organoids derived from normal tissues have a unique role in the study of liver physiology. The normal state of the hepatopancreatic islet axis is closely related to the normal functions of the liver and other organs of the mammalian body. Tao et al. (2021) simulated the human hepatopancreatic islet axis under normal conditions by generating a microfluidic multi-organ system that contained two separated regions connected by a network of microchannels. Due to the circulating perfusion conditions of this system, IPSC-derived liver and pancreatic organoids were able to persist in long-term culture.

Regenerative medicine is a field of medical research based on the natural healing ability of tissues that each organism possesses within itself. The liver, due to its powerful regenerative capacity, has opened up a wide range of prospects for the clinical application of hepatocyte transplantation and artificial liver generation using hepatocytes. Monogenic inherited liver diseases are caused by genetic defects in hepatocyte function in the absence of stem cell damage, and thus, liver transplantation can play an important role in the treatment of monogenic inherited diseases (Peng et al., 2021).

## 7 Conclusion and perspective

In summary, PSC- or ASC-derived liver organoids are gradually replacing cell lines and animal models as promising *in vitro* models for clinical studies of the liver due to their ability to better mimic the *in vivo* environment. Organoids have demonstrated their potential in modeling infectious, genetic, and alcoholic liver diseases, as well as in drug screening and personalized medicine.

IPSC-derived organoids have the advantage of not relying on primary tissue resection because patient-derived iPSC lines can generate multiple cell types indefinitely and repeatedly (Takebe et al., 2014). Unfortunately, there are genetic and epigenetic abnormalities associated with this process that are difficult to control. Thus, in disease modeling, patient biopsy-derived organoids may be superior to IPSC-derived organoids because they retain the patient’s true epigenetic characteristics, which are often greatly influenced by non-genetic environmental factors. Multiple tissue biopsies combined with PDO drug screening platforms can support drug efficacy studies, leading to individualized treatment (Li et al., 2019). Although the clinical availability of patient-derived biopsy tissues is a major shortcoming, the positive contributions to drug screening and toxicity evaluation following the application of organoid disease models may increase patient acceptance of biopsies in the future. In addition, organoids can be a valuable method for early-decision making in the development of expensive anti-tumour drugs (Bertolini et al., 2015). Regardless, the emergence of organoids has addressed ethical issues to some extent (Rossi et al., 2018), and thus, the potential of organoids in clinical settings in the future is enormous. We are excited to see more comprehensive development of organoids in the future, including the application of new biomaterials that can be constructed not only in basic research, but increasingly used in actual clinical treatments.
